# Exploring the competitive dynamic enzyme allocation scheme through enzyme cost minimization

**DOI:** 10.1038/s43705-023-00331-8

**Published:** 2023-11-20

**Authors:** Shanshan Qi, Gangsheng Wang, Wanyu Li, Shuhao Zhou

**Affiliations:** 1 State Key Laboratory of Water Resources Engineering and Management, Wuhan University, Wuhan, 430072 China; 2 Institute for Water-Carbon Cycles and Carbon Neutrality, School of Water Resources and Hydropower Engineering, Wuhan University, Wuhan, 430072 China

**Keywords:** Microbial ecology, Biogeochemistry

## Abstract

Enzyme allocation (or synthesis) is a crucial microbial trait that mediates soil biogeochemical cycles and their responses to climate change. However, few microbial ecological models address this trait, particularly concerning multiple enzyme functional groups that regulate complex biogeochemical processes. Here, we aim to fill this gap by developing a COmpetitive Dynamic Enzyme ALlocation (CODEAL) scheme for six enzyme groups that act as indicators of inorganic nitrogen (N) transformations in the Microbial-ENzyme Decomposition (MEND) model. This allocation scheme employs time-variant allocation coefficients for each enzyme group, fostering mutual competition among the multiple groups. We show that the principle of enzyme cost minimization is achieved by using the substrate’s saturation level as the factor for enzyme allocation, resulting in an enzyme-efficient pathway with minimal enzyme cost per unit metabolic flux. It suggests that the relative substrate availability affects the trade-off between enzyme production and metabolic flux. Our research has the potential to give insights into the nuanced dynamics of the N cycle and inspire the evolving landscape of enzyme-mediated biogeochemical processes in microbial ecological modeling, which is gaining increasing attention.

Microorganisms play primary roles in soil carbon (C) and nutrient cycling and exert a strong response to climate change [1]. Of particular interest is how to explicitly integrate the diverse and complex microbial communities and their functional traits into ecosystem models [2]. Enzyme groups have been advocated as proxies for soil functions in microbial ecological models [3, 4]. However, the internal mechanisms of the allocation (or synthesis) of multiple enzyme functional groups remains poorly understood.

The soil nitrogen (N) cycling presents an ideal case for testing these underlying mechanisms because the microbial processes and their enzyme functional groups are relatively well known [5]. This cycling includes N fixation, nitrification, and sequential denitrification processes, which are mediated by six respective enzyme groups, i.e., nitrogenases, ammonia oxidases, nitrate reductases, nitrite reductases, nitric oxide reductases, and nitrous oxide reductases [6]. Since these intracellular N enzyme groups are challenging to directly measure, their incorporation into microbial ecological models has received limited attention. Hence, a compelling and systematic allocation scheme for multiple enzyme groups is imperative to advance microbial ecological modeling, ensuring alignment with the rapid progress in microbial ecology concerning N cycling.

To this end, we implemented C-N coupled modeling using the Microbial-ENzyme Decomposition (MEND) model (Supplementary Fig. S1), as it applies a COmpetitive Dynamic Enzyme ALlocation (CODEAL) scheme to account for the synthesis of the aforementioned six N-enzyme groups [4]. Here, “dynamic” refers a time-variant allocation (or synthesis) of each enzyme group, while “competitive” means that multiple enzyme groups employ their allocation coefficients linked to the corresponding substrate availability, allowing them to compete with each other (Eq. S41). These N-enzyme groups catalyze the transformation of six inorganic N substrates: ammonium (NH_4_^+^), nitrate (NO_3_^–^), nitrite (NO_2_^–^), nitric oxide (NO), nitrous oxide (N_2_O), and dinitrogen (N_2_). Note that each N-enzyme group encompasses multiple enzymes catalyzing the same reaction. We employ Michaelis-Menten kinetics to describe these intracellular N enzymes-mediated reactions in a “pseudo-mechanistic” rather than “truly-mechanistic” manner, as it offers a manageable approach to model the intricate biological dynamics involved in inorganic N cycling [4]. More details on MEND model and its state variables, governing equations, component fluxes and parameters are described in Supplementary Section 1 and Tables S2–S6.

We investigated three distinct enzyme allocation scenarios, namely A0, A1, and A2 (formulas in Supplementary Table S1), to determine the allocation coefficients. The A0 scenario defines the allocation coefficient as ${N}_{i}/{\sum }_{j=1}^{6}{N}_{j}$ (Eq. S1), representing the proportion of an inorganic N substrate’s concentration (${N}_{i},i={{{{\mathrm{1,2}}}}},\cdots ,6$) to the total inorganic N substrates (${\sum }_{j=1}^{6}{N}_{j}$), with the substrate concentration (*N*_*i*_) as the weighting factor. In the A1 scenario, the weighting factor of each enzyme group is denoted by the saturation level of an inorganic substrate (${N}_{i}/{{KsN}}_{i}$), resulting in the A1 allocation coefficient as $({N}_{i}/{{KsN}}_{i})/{\sum }_{j=1}^{6}\left({N}_{j}/K{{sN}}_{j}\right)$ (Eq. S2), where *KsN*_*i*_ is the half-saturation constant of an inorganic N substrate as quantified in Zhu et al. [7]. Finally, the A2 scenario utilizes the inverse weighting factor of A1, i.e., ${{KsN}}_{i}{/N}_{i}$, which indicates the allocation coefficient of $({Ks}{N}_{i}/{N}_{i})/{\sum }_{j=1}^{6}\left({Ks}{N}_{j}/{N}_{j}\right)$ (Eq. S3). We base the A2 scenario on an evolutionarily stable strategy, implying an optimal growth rate for a specific habitat [8]. Consequently, microbes may adjust their metabolic activities to achieve optimal growth and ensure balanced flux across multiple processes within a given condition. This scenario attempts to apply biochemical kinetics to represent a potential mechanism that governs the distribution of cellular resources over processes to maintain a stable flux [9]. The A0 and A1 scenarios imply that a higher amount of enzymes will be produced to consume the corresponding substrate under a higher substrate concentration or saturation level. On the contrary, A2 is expected to produce fewer enzymes to maintain a stable flux, given a lower weighting factor of ${{KsN}}_{i}{/N}_{i}$. This corresponds to a higher substrate saturation level, as expressed by ${N}_{i}/({N}_{i}+{{KsN}}_{i})$ in the Michaelis-Menten (M-M) kinetics [10, 11].

We hypothesized that an optimal allocation strategy for multiple enzyme groups is to utilize the relative saturation level of a substrate as the competitive dynamic allocation coefficient (i.e., the A1 scenario), which enables the establishment of enzyme-efficient metabolic pathways while minimizing enzyme costs. Following the recent study by Wang et al. [4] that comprehensively calibrated diverse soil C-N fluxes from a 12-year CO_2_×N grassland experiment (BioCON), we conducted the three allocation scenarios through fitting the observed inorganic N pools (NH_4_^+^ and NO_3_^–^ + NO_2_^–^) and fluxes (biological N fixation (BNF), net N mineralization, nitrification, and plant N uptake) (Supplementary Table S7).

The A1 scenario outperforms both A0 and A2, particularly for the BNF flux (Fig. 1a) and the plant N uptake flux (Fig. 1b). All three scenarios simulate the observed N pools (NH_4_^+^ and NO_3_^–^ + NO_2_^–^) and the other two N fluxes (net N mineralization and nitrification) well, similar to the results in Wang et al. [4] (Table S7). Among these scenarios, A1 emerges as the optimal choice, producing the maximum total inorganic N flux (Fig. 1c) with the minimum amount of enzyme (Fig. 1d), which embodies the theory of enzyme cost minimization [12]. This principle has been studied empirically on ecoenzyme production and activity [13, 14] or theoretically based on metabolic flux and kinetic models [12]. However, very few studies have adopted this principle within microbial ecological modeling. Natural selection has been shown to favor the production of enzymes that balance the costs and benefits [13]. Here, A1 effectively maximizes benefits with enzyme cost minimization, which is more likely to be selected. Continuous efforts have been devoted to the theoretical analysis of enzyme allocation schemes [15–17]. These analyses underscore the importance of enzyme allocation in relation to metabolic requirements and substrate availability, which must be considered in the context of soil C and nutrient dynamics [18]. However, there is currently a gap in understanding and modeling the allocation strategy for multiple enzyme groups, particularly those involving more than three enzyme groups. Furthermore, few microbial ecological models explicitly address this microbial trait, i.e., enzyme allocation, in complex N transformation processes. N availability significantly affects microbial growth and biogeochemical cycles [6]. This study marks an initial endeavor to propose an effective enzyme allocation strategy, in alignment with the enzyme cost minimization theory, for multiple enzyme groups within C-N coupled ecological models.

**Fig. 1:**
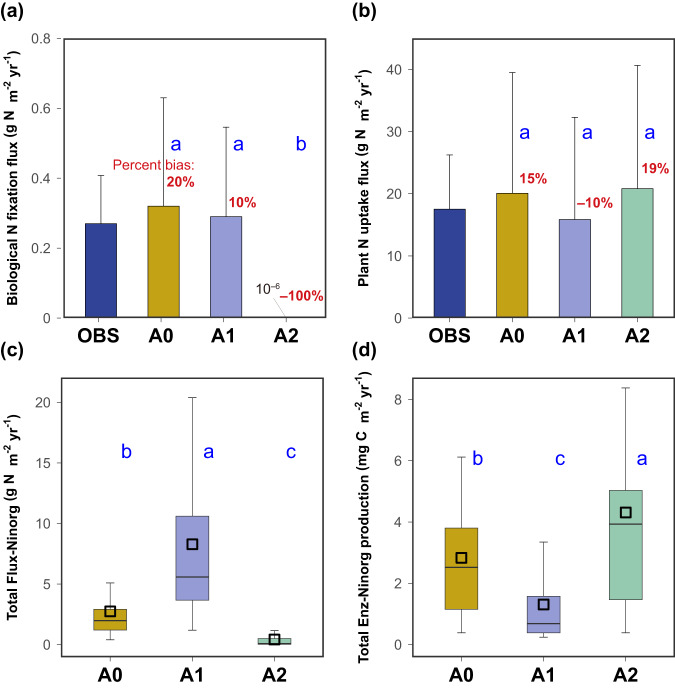
Three enzyme allocation scenarios (A0, A1, and A2) to produce six enzyme groups catalyzing inorganic nitrogen (N) transformations in the Microbial-ENzyme Decomposition (MEND) model. **a**, **b** Comparison among simulated (A0, A1, and A2) and observed (OBS) biological N fixation flux and plant N uptake flux, respectively. **c**, **d** Total inorganic N flux (Flux-Ninorg) and total production of enzymes operating on inorganic N (Enz-Ninorg) as per the three scenarios. Flux-Ninorg is the sum of the fluxes of biological N fixation, nitrification and denitrification processes. Enz-Ninorg includes six enzyme groups catalyzing inorganic N cycling, i.e., nitrogenases, ammonia oxidases, nitrate reductases, nitrite reductases, nitric oxide reductases, and nitrous oxide reductases. Different letters (a, b, and c) denote significant difference (*p-*value < 0.001) by the Wilcoxon signed rank test. Error bars in **a**, **b** are standard deviations (*n* = 24). Percentage labeled in red denotes the percent bias between simulated and observed average values.

These enzymes that operate on inorganic N are either membrane-bound or located in the cell cytoplasm and periplasm [19, 20]. Intracellular enzyme activity is likely to more immediately respond to substrate availability than that of extracellular enzymes [21]. Based on the empirical relationships, the three allocation scenarios establish distinct linkages between them. The comparison between A0 and A1 highlights that incorporating enzymatic kinetic parameter (half-saturation constant, *Ks*) fosters a more efficient metabolic pathway. However, A2 notably underperforms, particularly in the significant underestimation of the BNF flux (Fig. 1a), as indicated by the percent bias (see Eq. S58), which represents the relative error as a percentage between the simulated and observed mean values. Dinitrogen (N_2_) is the largest freely available N reservoir within terrestrial ecosystems [22]. This translates to a notable decrease in nitrogenases production in A2, while A0 and A1 exhibit elevated enzyme level (Fig. 1a). Additionally, the BNF flux is inhibited by a higher soil ammonium availability (i.e., the saturation level of NH_4_^+^) within the MEND model [4, 23] (Eq. S46). We observed a consistent rise in NH_4_^+^ availability within A2 (Supplementary Fig. S3c), exacerbating the decline of the BNF flux to extremely low levels. This further suppresses the amount of ammonia oxidases and sequential products, favoring increased N-reductases allocation (Fig. 1d). To better understand the variation in outcomes of the three allocation scenarios, we quantified the uncertainty in modeling the N-dynamics component, specifically the total inorganic N flux (Flux-Ninorg) and the total production of N-related enzymes (Enz-Ninorg), in response to variations in N-relevant model parameters (see methods in Supplementary Section 3.2). The relative uncertainty (ReUn = Width_90%CI_/Mean, see Eq. S62 and ref. [24]) of Flux-Ninorg in A1 has a mean value of 10% (range: 4–14%), which is much lower than the ReUn in A0 (25% with a range of 5–38%) and A2 (36% with range of 9–64%) (Supplementary Fig. S4a). Enz-Ninorg also exhibits the lowest ReUn in A1 (5%) compared to that of A0 (8%) and A2 (18%) (Fig. S4b). These results indicate that, in addition to its enzyme-efficient behavior, A1 could achieve more robust results than A0 and A2. We acknowledge that microbial physiology in the real world is likely more intricate than what modeling can capture [16, 25]. For example, microbial communities may possess the specific ability to regulate excessive or insufficient enzyme production and sustain flux equilibrium [9]. The A2 scenario described here may not be physically realistic in achieving this goal.

In summary, we introduce the CODEAL, an innovative enzyme allocation scheme for governing multiple enzyme groups under dynamic substrate concentrations in microbial ecological modeling. CODEAL has been robustly tested within the MEND model, designed for modeling microbial-enzyme-driven C-N coupled dynamics. We advocate using relative substrate availability as the weighting factor for enzyme allocation, as it yields an enzyme-efficient pathway that maximizes returns on investment (enzyme production). Our findings highlight the significant influence of relative substrate availability on the trade-off between enzyme production and metabolic flux, a crucial consideration in simulations of enzyme-mediated biogeochemical processes. Furthermore, these results may illuminate the intricate mechanisms that govern diverse N cycling processes carried out by metabolically versatile soil microorganisms.

### Supplementary information

Supplementary material

## Data Availability

The data described in this article are openly available from: https://zenodo.org/records/10050714.
