# Supplementary material for: Exploring the competitive dynamic enzyme allocation scheme through enzyme cost minimization
Source: ISME Commun. 2023 Nov 20;3:121. doi: 10.1038/s43705-023-00331-8 (PMC10662282; doi:10.1038/s43705-023-00331-8)
Supplement: Supplementary file 1 — Supplementary material [file 43705_2023_331_MOESM1_ESM.docx]

**Supplementary material**

**Exploring the competitive dynamic enzyme allocation scheme through enzyme cost minimization**

Shanshan Qi^1,2^, Gangsheng Wang^1,2,*^, Wanyu Li^1,2^, Shuhao Zhou^1,2^

^1^State Key Laboratory of Water Resources Engineering and Management, Wuhan University, Wuhan, 430072, China

^2^Institute for Water-Carbon Cycles and Carbon Neutrality, School of Water Resources and Hydropower Engineering, Wuhan University, Wuhan, 430072, China

^*^Corresponding author: Gangsheng Wang, *E-mail address*: [wanggs@whu.edu.cn](mailto:wanggs@whu.edu.cn)

# 1. Methods and materials

## 1.1 Carbon-Nitrogen (C-N) coupled MEND model

The Microbial-Enzyme Decomposition (MEND) model allows for mechanistic representation of C-N dynamic processes using separate microbial and enzymatic groups [1]. The model includes several SOM (with both C and N) pools that differ by their density and physicochemical protection.

Key features are: first, distinct enzyme functional groups regulate SOM decomposition and inorganic N transformations processes (i.e., N mineralization & immobilization, biological N fixation, nitrification, and sequential denitrification). Second, the adaption of microbes to stoichiometric imbalances applies a time-variant C/N ratio [2-4] except for the enzyme pools, where a fixed C/N (= 3) is used [5, 6]. Besides, the representation of microbial physiology (e.g., growth and maintenance, dormancy and resuscitation, and mortality) which shows changes in soil pH, temperature, and moisture [7-9]. Finally, the depiction of plant-microbial competition for inorganic N (NH_4_^+^ and NO_3_^–^), ammonium (NH_4_^+^) sorption and nitrate (NO_3_^–^) & nitrite (NO_2_^–^) leaching, and N gases (NO, N_2_O, and N_2_) exchange between soil and the atmosphere. Model state variables, governing equations, component fluxes and parameters are described in Supplementary Table S2–S6.


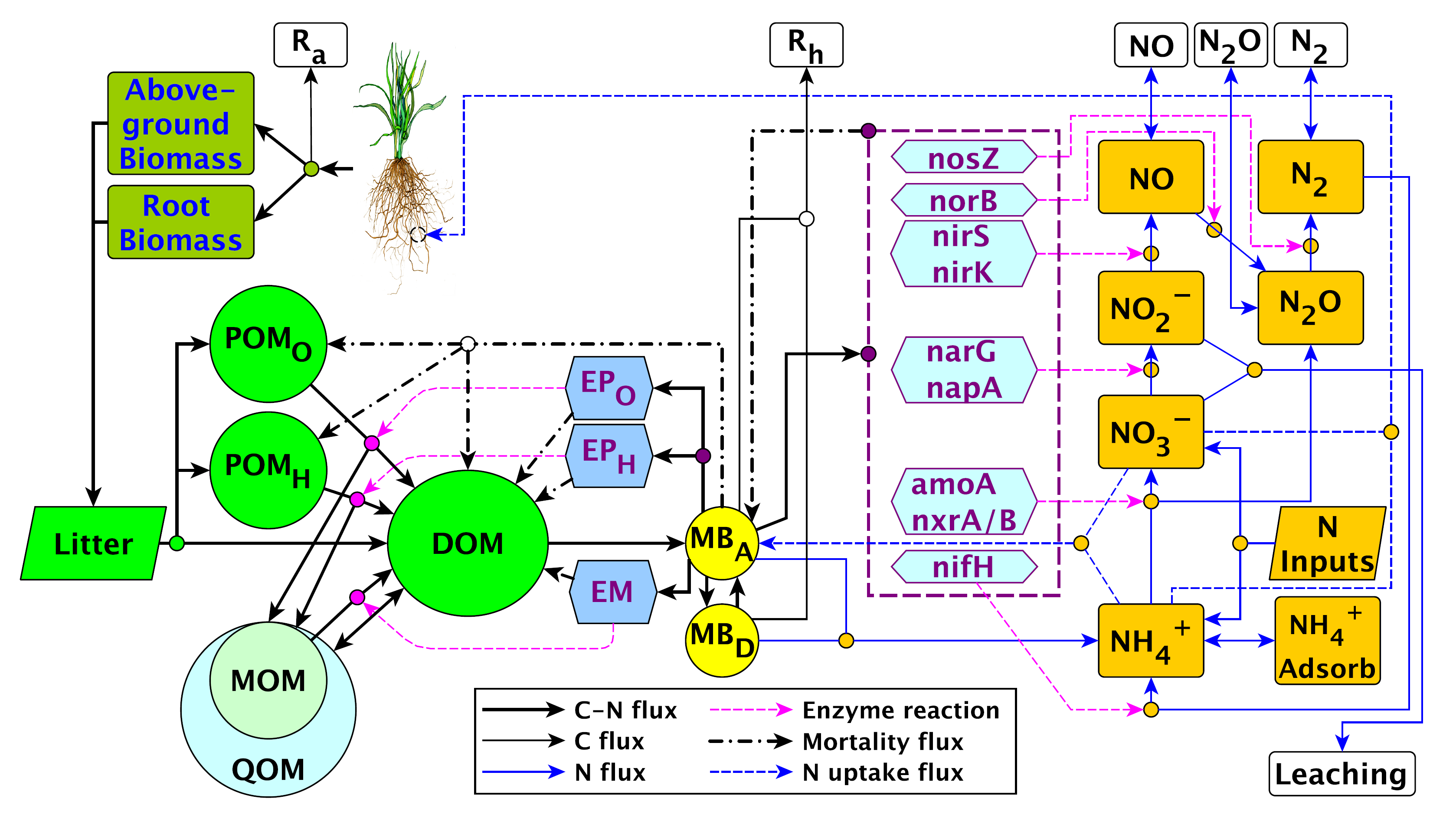


**Figure S1. Diagram of the Microbial-ENzyme Decomposition (MEND) model.** R_a_ and R_h_ are autotrophic and heterotrophic respiration, respectively. POM_O_ and POM_H_ are particulate organic matter (POM) decomposed by oxidative (EP_O_) and hydrolytic enzymes (EP_H_), respectively. MOM is mineral-associated OM, which is decomposed by a mixed enzyme group EM. Dissolved OM (DOM) interacts with the active layer of MOM (QOM) through sorption and desorption. Litter enters POM_O_, POM_H_, and DOM. Microbes consist of active (MB_A_) and dormant microbes (MB_D_). DOM can be assimilated by MB_A_. Inorganic N deposition and fertilization enter NH_4_^+^ and NO_3_^–^ that can be immobilized by microbes and taken up by plant roots. NH_4_^+^ adsorption is also considered. N fixation, nitrification and denitrification are mediated by nitrogenases (*nifH*), ammonia oxidases (*amoA*, *nxrA/B*) and N-reductases (*narG/napA*, *nirS/nirK*, *norB*, *nosZ*), respectively. Inorganic N loss pathways include leaching (NO_3_^–^ and NO_2_^–^) and gas emission (NO, N_2_O, and N_2_) from the soil to the atmosphere.

## 1.2 COmpetitive Dynamic Enzyme ALlocation (CODEAL) scheme

The MEND model implements a CODEAL scheme to deal with the allocation of multiple enzyme groups, according to the synthetic results that enzyme activities are dependent on microbial biomass [10] and substrate availability [11]. The synthesis of SOM-degrading enzymes depends upon the active microbial biomass and the relative abundance of the C substrate that needs to be decomposed (Eq. S39) [12]. The total synthesis rate of all inorganic-N enzyme groups is assumed to be proportional to the total synthesis rate of all SOM-degrading enzymes by a scaling factor, which is the ratio of total soil inorganic N to total soil organic N (Eq. S41). The turnover of SOM-degrading enzymes enters the dissolved organic matter (DOM) pool (Eq. S40), whereas the turnover of six inorganic-N enzyme groups becomes microbial biomass [13-15].

We propose three distinct enzyme allocation scenarios, namely A0, A1, and A2, to determine the competitive allocation coefficients for inorganic-N transformation processes (Table S1). The six inorganic-N enzyme groups are all intracellular enzymes that are either membrane-bound or located in the cell cytoplasm and periplasm. Since they have little ability to live on their own and are less affected by the external environment, their expression process strongly depends on substrate availability [11, 16].

## 1.3 BioCON datasets for model parameterization and calibration

The BioCON (Biodiversity, CO_2_, and N deposition) datasets provide well-designed, long-term multifactor free-air CO_2_ enrichment experiments [17]. MEND utilizes nine C-N response variables for model parameterization and calibration [1]: soil CO_2_ flux (Rs), microbial biomass C (MBC), soil organic C (SOC), ammonium (NH_4_^+^), nitrate + nitrite (NO_3_^–^+NO_2_^–^), net N mineralization, nitrification, biological N fixation (BNF), and plant N uptake. We conducted tests on the three enzyme allocation scenarios through fitting the observed last six variables. The performance for three scenarios is shown in Table S7. MEND incorporates the modified Shuffled Complex Evolution (SCE) method [18, 19] to facilitate model calibration against user-selected variables, with the aim of minimizing the overall objective function denotes as “$J$” (see Supplementary Section 1.4). SCE is a stochastic optimization algorithm that includes competitive evolution of a set of points spanning the parameter space and the shuffling of complexes, which has been widely used in calibration of hydrological and ecological models and has proved to be efficient and robust [20, 21].

Soil CO_2_ flux (soil respiration) in each plot was measured from 11 to 36 times per year using a LI-COR 6400-09 soil respiration chamber (LI-COR, Lincoln, Nebraska, USA). There were 284 time points of soil respiration fluxes for each treatment from 1998 to 2009. Plant C/N ratio (aboveground plant and root) and soil inorganic N pools and fluxes were measured in July–August of each year [22]. Soil NH_4_^+^ and NO_3_^–^ in four removed soil cores were extracted with 1 M KCl and their concentrations were measured on an Alpkem autoanalyzer (OI Analytical, College Station, Texas, USA). Net N mineralization rates were calculated as the difference between the total inorganic N (NH_4_^+^ and NO_3_^–^) in the field-incubated PVC tubes and that in the soil cores removed roughly one month earlier. Net nitrification rates were estimated using only NO_3_^–^ data [23]. In this study, the model achieved good performance in simulating soil respiration fluxes among different scenarios (Fig. S2a), comparable to the result in Wang et al. [1]. The simulated soil NH_4_^+^ and (NO_3_^−^ + NO_2_^−^) concentrations also agreed well with the observations (Fig. S2b).

**
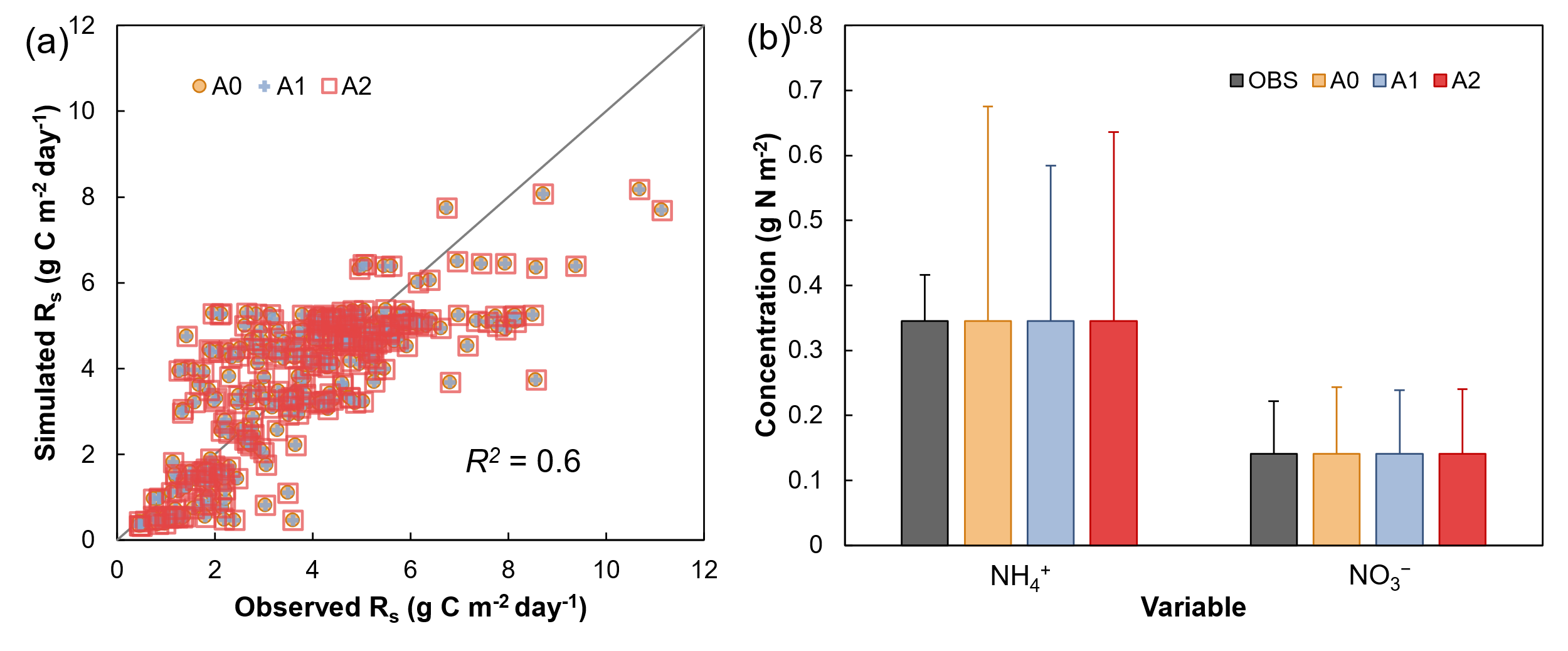
**

**Figure S2.** **Model calibration results.** (a) Soil respiration (R_s_). (b) Soil ammonium (NH_4_^+^) and nitrate (NO_3_^−^, including both NO_3_^−^ and NO_2_^−^) concentrations. *R^2^* value in (a) denotes the coefficient of determination. Error bars in (b) are standard deviations (n = 8).

## 1.4 Multi-objective Functions for evaluating model performance

We employ multi-objective functions to judge the performance of different scenarios [12, 20]. Model calibration aims to minimize the overall objective function value (*J*) that is computed as the weighted average of multiple single-objectives [24]:

| $J=\sum_{i=1}^{m} w_{i}\cdot J_{i}$ | (S56a) |
| --- | --- |
| $\sum_{i=1}^{m} w_{i}=1$ and $w_{i}\in[0,1]$ | (S56b) |

where *m* denotes the number of objectives and *w_i_* is the weighting factor for the *i*^th^ (*i* = 1, 2, … , *m*) objective function (*J_i_*).

We select four objective functions ($R^{2}$, |*PBIAS*|, *MARE*, and *MAREt*) to assess the modeling performance:

| $R^{2}=1-\frac{\sum_{i=1}^{n} \left[ Y_{sim}\left( i \right)-Y_{obs}\left( i \right) \right]^{2}}{\sum_{i=1}^{n} \left[ Y_{obs}\left( i \right)-\bar{Y}_{obs} \right]^{2}}$ | (S57) |
| --- | --- |
| $\vert PBIAS\vert=\left\vert\frac{\bar{Y}_{sim}-\bar{Y}_{obs}}{\bar{Y}_{obs}} \right\vert$ | (S58) |
| $MARE=\frac{1}{n}\sum_{i=1}^{n} \left\vert\frac{Y_{sim}\left( i \right)-Y_{obs}\left( i \right)}{Y_{obs}\left( i \right)} \right\vert$ | (S59) |
| $MAREt=\left\{ \begin{aligned} 0, &MARE\leq tolerance \\ MARE, &MARE>tolerance \end{aligned} \right.$ | (S60) |

where *R*^2^ denotes the coefficient of determination; |*PBIAS*| is the percent bias between simulated and observed mean values; *MARE* is the Mean Absolute Relative Error (MARE) and represents the averaged deviations of simulations (*Y_sim_*) from their observations (*Y_obs_*); *MAREt* is a variant of *MARE* and *MAREt* achieves the best (= 0) when *MARE* is within a defined tolerance value; *n* is the number of data; *Y_obs_* and *Y_sim_* are observed and simulated values, respectively; and $\bar{Y}_{obs}$ and $\bar{Y}_{sim}$ are the mean value for *Y_obs_* and *Y_sim_*, respectively.

Different objective functions are employed to quantify the goodness-of-fit for different variables, depending on the measurement method and frequency of variables. *R*^2^ quantifies the proportion of the variance in the response variables that is predictable from the independent variables [7]. A higher *R*^2^ (≤ 1) indicates better model performance. *R*^2^ is used to evaluate the variables (e.g., total soil respiration or heterotrophic respiration) that are frequently measured, and the absolute values can be directly compared between observations and simulations. *MARE* or |*PBIAS*| is used to evaluate the variables with only a few measurements (e.g., concentrations of inorganic N) and the absolute values can be directly compared. Lower *MARE* or |*PBIAS*| values (≥ 0) are preferred [21]. *MAREt* should be used when the simulated value may not be necessarily to strictly match the observed or measured value. For example, the measured nitrification rates are more like potential rates or rough estimates. It is more appropriate to use *MAREt* to evaluate the model simulated actual nitrification rates when compared with these measurements. Another example includes the comparison between simulated and observed N fixation [25] or plant N uptake rates [26]. The observations collected from literature just represent empirical or reference N fixation or plant N uptake rates. In this case, we also recommended to use *MAREt* as we expected the simulated N fixation or plant N uptake rates would fall into the observed value ranges.

# 2. Supplementary tables

## Table S1. Three competitive dynamic allocation scenarios

| Allocation scenarios | Competitive allocation coefficients | Eq# |
| --- | --- | --- |
| A0, the corresponding inorganic N substrate | $N_{i}/\sum_{j=1}^{6} N_{j}$ | (S1) |
| A1, the relative saturation level of a substrate | $(N_{i}/{KsN}_{i})/\sum_{j=1}^{6} \left( {N_{j}}/{K{sN}_{j}} \right)$ | (S2) |
| A2, the inverse weight of A1 | $(KsN_{i}/N_{i})/\sum_{j=1}^{6} \left( Ks{N_{j}}/{N_{j}} \right)$ | (S3) |

**Note:** $N_{i}$ denotes the specific inorganic N substrate: ammonium (NH_4_^+^), nitrate (NO_3_^–^), nitrite (NO_2_^–^), nitric oxide (NO), nitrous oxide (N_2_O), and dinitrogen (N_2_); $KsN_{i}$ is the half-saturation constant of the specific inorganic N substrate; $\sum_{j=1}^{6} N_{j}$ is the total amount of inorganic N substrates.

## Table S2. Soil carbon (C) and nitrogen (N) pools (state variables) in the MEND model

| **Soil C and/or N pool** | **Pool name** | **Description** (variable name) |
| --- | --- | --- |
| SOM pools | POM_O_ | Particulate organic matter (POM) decomposed by oxidative enzymes (*PO; PON*) |
|  | POM_H_ | POM decomposed by hydrolytic enzymes (*PH; PHN*) |
|  | MOM | Mineral-associated organic matter (*M; MN*) |
|  | DOM | Dissolved organic matter (*D; DN*) |
|  | QOM | Active MOM interacting with DOM (*Q; QN*) |
| SOM-degrading enzyme groups | EP_O_ | POM oxidative enzymes (*EPO; EPON*) |
|  | EP_H_ | POM hydrolytic enzymes (*EPH; EPHN*) |
|  | EM | MOM degrading enzymes (*EM; EMN*) |
| Inorganic-N enzyme groups | ENH4 | Ammonium oxidases (*ENH4; ENH4N*) |
|  | ENO3 | Nitrate reductase (*ENO3*; *ENO3N*) |
|  | ENO2 | Nitrite reductases (*ENO2; ENO2N*) |
|  | ENO | Nitric oxide reductases (*ENO; ENON*) |
|  | EN2O | Nitrous oxide reductases (*EN2O; EN2ON*) |
|  | EN2 | Nitrogenases (*EN2; EN2N*) |
| Inorganic N pools | NH_4_^+^ Adsorb | Adsorbed ammonium (*NH4ads*) |
|  | NH_4_^+^ | Ammonium (*NH4*) |
|  | NO_3_^–^ | Nitrate (*NO3*) |
|  | NO_2_^–^ | Nitrite (*NO2*) |
|  | NO | Nitric oxide (*NO*) |
|  | N_2_O | Nitrous oxide (*N2O*) |
|  | N_2_ | Dinitrogen (*N2*) |
| Microbial functional groups | MB_A_ | Active microbial biomass (*BA; BAN*) |
|  | MB_D_ | Dormant microbial biomass (*BD; BDN*) |

## Table S3. Governing equation for each soil C or N pool (Table S2) in the MEND model

| **Governing Equation** | **Eq#** |
| --- | --- |
| *Soil Carbon* | |
| ${dPO}/{dt}=I_{PO}+\left( 1-g_{D} \right)\cdot g_{PO}\cdot F_{9} F_{1}$; $I_{PO}+I_{PH}+I_{D}=I_{gross}\cdot fINP$; $I_{gross}$ is gross litter input | (S4) |
| ${dPH}/{dt}=I_{PH}+\left( 1-g_{D} \right)\cdot\left( 1-g_{PO} \right)\cdot F_{9} F_{2}$ | (S5) |
| ${dM}/{dt}=\left( 1-f_{D} \right)\cdot\left( F_{1}+F_{2} \right) F_{3}$ | (S6) |
| ${dQ}/{dt}=F_{4}-F_{5}$ | (S7) |
| ${dD}/{dt}=I_{D}+f_{D}\cdot\left( F_{1}+F_{2} \right)+ F_{3}+g_{D}\cdot F_{9}+F_{16}-F_{6}-(F_{4}-F_{5})$ | (S8) |
| ${dBA}/{dt}=F_{6}-\left( F_{7}-F_{8} \right)-F_{9}-\left( F_{10}+F_{11}+F_{12} \right)-\left( F_{15}+F_{17} \right)+F_{18}$ | (S9) |
| ${dBD}/{dt}=\left( F_{7}-F_{8} \right){-(F}_{13}+F_{14})$ | (S10) |
| ${dED_{i}}/{dt}=F_{15,ED_{i}}-F_{16,ED_{i}}$; $ED_{i}$ ($i=1,2,3$) denotes *EPO*, *EPH*, *EM*, respectively | (S11) |
| ${dEN_{j}}/{dt}=F_{17,EN_{j}}-F_{18,EN_{j}}$; $EN_{j}$ ($j=1,2,\cdots,6$) denotes *ENH4*, *ENO3*, *ENO2*, *ENO*, *EN2O*, *EN2*, respectively | (S12) |
| $R_{h}=\left( F_{10}+F_{11}+F_{12} \right)+(F_{13}+F_{14})$; heterotrophic (microbial) respiration  $R_{a}=fR_{a}\cdot GPP$; autotrophic (root) respiration  ${R_{s}=R}_{a}+R_{h}$; total soil respiration | (S13a)  (S13b)  (S13c) |
| $\frac{d}{dt}\left( PO+PH+M+Q+D+BA+BD+\sum_{i=1}^{3} ED_{i}+\sum_{j=1}^{6} EN_{i} \right)=\left( I_{PO}+I_{PH}+I_{D} \right)-R_{h}$ | (S14) |
| *Soil Nitrogen* | |
| - For soil organic matter pools, the *N* flux: $FN_{k}={F_{k}}/{CN_{source}}$, where *F_k_* (*k* = 1–9) is the *C* flux, and *CN_source_* is the C:N ratio of the (upstream) source pool. - For enzymes pools *ED_i_* (*i*=1,2,3) and *EN_j_* (*j*=1…6), the *N* flux ${FN}_{k}={F_{k}}/{CN_{ENZ}}$, *k* = 15–18. | (S15a)  (S15b) |
| $\frac{dBAN}{dt}=\frac{F_{6}}{CN_{D}}-\left( \frac{F_{7}}{CN_{BA}}-\frac{F_{8}}{CN_{BD}} \right)-\frac{F_{9}}{CN_{BA}}-\frac{F_{15}+F_{17}}{CN_{ENZ}}+\frac{F_{18}}{CN_{ENZ}}-FN_{mn,BA}+\left( FN_{im,NH4\to BA}+{FN}_{im, NO3\to BA} \right)$ | (S16) |
| ${dBDN}/{dt}=\left( F_{7}/CN_{BA}-F_{8}/CN_{BD} \right)-FN_{mn,BD}$ | (S17) |
| ${dNH4}/{dt}=I_{NH4}+FN_{fix}+\left( {FN}_{mn, BA}+{FN}_{mn, BD} \right)-\left( {FN}_{im, NH4\to BA}+{FN}_{im, NH4\to VG} \right)-FN_{nit}$ | (S18) |
| ${dNO3}/{dt}=I_{NO3}+FN_{nit}-FN_{nit-denit}-FN_{denit_{2}}-\left( {FN}_{im, NO3\to BA}+{FN}_{im, NO3\to VG} \right)-FN_{leach,NO3}$ | (S19) |
| ${dNO2}/{dt}=FN_{denit_{2}}-FN_{denit_{3}}-FN_{leach,NO2}$ | (S20) |
| ${dNO}/{dt}=FN_{denit_{3}}-FN_{denit_{4}}-FN_{emit_{4}}$ | (S21) |
| ${dN2O}/{dt}=FN_{nit-denit}+FN_{denit_{4}}-FN_{denit_{5}}-FN_{emit_{5}}$ | (S22) |
| ${dN2}/{dt}=FN_{denit_{5}}-FN_{fix}-FN_{emit_{6}}$ | (S23) |
| $\frac{d}{dt}\left( PON+PHN+MN+QN+DN+BAN+BDN+\sum_{i=1}^{3} E{DN}_{i}+\sum_{j=1}^{6} E{NN}_{i} \right)=(IN_{PO}+IN_{PH}+IN_{D})+\left( {FN}_{im, NH4\to BA}+{FN}_{im, NO3\to BA} \right)-\left( {FN}_{mn, BA}+{FN}_{mn, BD} \right)$ | (S24a) |
| $\frac{d}{dt}\left( NH4ads+\sum_{j=1}^{6} N_{j} \right)=\left( I_{NH4}+I_{NO3} \right)+\left( {FN}_{mn, BA}+{FN}_{mn, BD} \right)-\left( {FN}_{im, NH4\to BA}+{FN}_{im, NO3\to BA} \right)-\left( {FN}_{im, NH4\to VG}+{FN}_{im, NO3\to VG} \right)-\left( FN_{leach,NO3}+FN_{leach,NO2} \right)-\sum_{j=4}^{6} {NF}_{emit_{j}}$ | (S24b) |
| $\frac{d}{dt}\left( PON+PHN+MN+QN+DN+BAN+BDN+\sum_{i=1}^{3} E{DN}_{i}+\sum_{j=1}^{6} E{NN}_{i}+\sum_{j=1}^{6} N_{j}+NH4ads \right)=\left( {IN}_{PO}+{IN}_{PH}+{IN}_{D})+{(I}_{NH4}+I_{NO3} \right)-\left( {FN}_{im, NH4\to VG}+{FN}_{im, NO3\to VG} \right)-\left( FN_{leach,NO3}+FN_{leach,NO2} \right)-\sum_{j=4}^{6} {NF}_{emit_{j}}$ | (S24c) |

**Note**: Eqs. S14, S24a, S24b and S24c express the overall mass balance of soil organic C (SOC), soil organic N (SON), inorganic N and total N, respectively. The transformation fluxes (*F* or *FN*) are described in Table S4 and Table S5.

## Table S4. Component fluxes in the MEND model (parameters are shown in Table S6)

| **Flux description** | **Equation** | **Eq#** |
| --- | --- | --- |
| Particulate organic matter (POM) pool (oxidative) (*PO*) decomposition (*F*_1_) | $F_{1}={Vd_{PO}\cdot EPO\cdot PO}/\left( K_{PO}+PO \right)$ | (S25) |
| POM pool (hydrolytic) (*PH*) decomposition | $F_{2}={Vd_{PH}\cdot EPH\cdot PH}/\left( K_{PH}+PH \right)$ | (S26) |
| Mineral-associated organic matter (*M*) decomposition | $F_{3}={Vd_{M}\cdot EM\cdot M}/\left( K_{M}+M \right)$ | (S27) |
| Adsorption (*F*_4_) and desorption (*F*_5_) between dissolved organic matter (*D*) and adsorbed DOM (*Q*) | $F_{4}=k_{ads}\cdot(1-Q/Q_{max})\cdot D$  $F_{5}=k_{des}\cdot(Q/Q_{max})$  $K_{ads}=K_{des}\cdot K_{ba}$ | (S28)  (S29) |
| DOM (*D*) uptake by microbes | $F_{6}=\frac{1}{Y_{g}}\left( V_{g}+V_{m} \right)\cdot\frac{BA\cdot D}{K_{D}+D}$ | (S30) |
| Dormancy (*F*_7_) and resuscitation (*F*_8_) between active (*BA*) and dormant (*BD*) microbes | $F_{7}=\left[ 1-D/(K_{D}+D) \right]\cdot V_{m}\cdot BA$  $F_{8}=\left[ D/(K_{D}+D) \right]\cdot V_{m}\cdot BD$ | (S31)  (S32) |
| MB_A_ (*BA*) mortality | $F_{9}=\gamma\cdot V_{m}\cdot BA$ | (S33) |
| MB_A_ (*BA*) growth respiration (*F*_10_) and maintenance respiration (*F*_11_) | $F_{10}=\left( \frac{1}{Y_{g}}-1 \right)\cdot\frac{V_{g}\cdot BA\cdot D}{K_{D}+D}$  $F_{11}=\left( \frac{1}{Y_{g}}-1 \right)\cdot\frac{V_{m}\cdot BA\cdot D}{K_{D}+D}$ | (S34)  (S35) |
| MB_A_ (*BA*) overflow respiration (*F*_12_) | $F_{12}=max\left\{ 0,BA-BAN\cdot CN_{BA,max} \right\}$ | (S36) |
| MB_D_ (*BD*) maintenance respiration (*F*_13_) | $F_{13}=\beta\cdot V_{m}\cdot BD$ | (S37) |
| MB_D_ (*BD*) overflow respiration (*F*_14_) | $F_{14}=max\left\{ 0,BD-BDN\cdot CN_{BA,max} \right\}$ | (S38) |
| Synthesis of enzymes for decomposition of *PO* (*F*_15,EPO_, *EPO* = *ED*_1_), *PH* (*F*_15,EPH_, *EPH* = *ED*_2_), and *M* (*F*_15,EM_, *EM* = *ED*_3_) | $F_{15,EPO}={PO/(PO+PH)\cdot p}_{EP}\cdot V_{m}\cdot BA$  $F_{15,EPH}={PH/(PO+PH)\cdot p}_{EP}\cdot V_{m}\cdot BA$  $F_{15,EM}={fp_{EM}\cdot p}_{EP}\cdot V_{m}\cdot BA$  $F_{15}=\sum_{i=1}^{3} F_{15,ED_{j}}=F_{15,EPO}+F_{15,EPH}+F_{15,EM}$ | (S39) |
| Turnover of enzymes (*EPO* = *ED*_1_, *EPH* = *ED*_2_, *EM* = *ED*_3_) | $F_{16,ED_{i}}=r_{E}\cdot ED_{i}$  $F_{16}=\sum_{i=1}^{3} F_{16,ED_{j}}$ | (S40) |
| Synthesis of enzyme groups for inorganic-N transformations;  $EN_{j}$ ($j=1-6$): *ENH4*, *ENO3*, *ENO2*, *ENO*, *EN2O*, *EN2*;  ${SON}_{i}$ ($i=1-5$): *PON*, *PHN*, *MN*, *QN*, *DN* | $F_{17}={\sum_{j=1}^{6} F_{17,EN_{j}}=F}_{15}\cdot\left( NH4+NO3+NO2 \right)/{\sum_{i=1}^{4} {SON}_{i}}$  $F_{17,EN_{j}}=ALCOE\cdot F_{17}$ | (S41) |
| Turnover of enzymes for nitrification, denitrification, and N fixation | $F_{18}=\sum_{j=1}^{6} F_{18,EN_{j}}$; $F_{18,EN_{j}}=r_{E}\cdot EN_{j}$ | (S42) |

**Note**: ‘*ALCOE*’ in Eq. S41 denotes the competitive allocation coefficient described in Table S1.

## Table S5. Inorganic N fluxes in the MEND model (parameters are shown in Table S6)

| **Flux description** | **Equation** | **Eq#** |
| --- | --- | --- |
| Nitrification | $FN_{nit}=\frac{VN_{nit}\cdot ENH4\cdot NH4}{KSN_{1}+NH4}$ | (S43) |
| Nitrifier Denitrification | $FN_{nit-denit}=FN_{nit}\cdot\left[ 1-f{(O}_{2}) \right]$  $f\left( O_{2} \right)=\frac{\left( 1-WFP \right)^{4/3}}{{0.5}^{4/3}+\left( 1-WFP \right)^{4/3}}$; *WFP* is water-filled porosity | (S44a)  (S44b) |
| Denitrification of *NO3*, *NO2*, *NO*, *N2O*: *j* = 2, 3, 4, 5 | ${FN}_{denit_{j}}=\frac{VN_{j}\cdot{EN_{j}\cdot N}_{j}}{KSN_{j}+N_{j}}$ | (S45) |
| N fixation | $FN_{fix}=\frac{VN_{fix}\cdot EN2\cdot N2}{KSN_{6}+N2}\cdot\left( 1-\frac{NH4}{KSN_{1}+NH4} \right)$ | (S46) |
| N mineralization | $FN_{mn,BA}=(1-{YN}_{g})\cdot{FN}_{6}$  $YN_{g}=\left( \frac{CN_{BA}-CN_{BA,min}}{CN_{BA,max}-CN_{BA,min}} \right)^{\omega}$ | (S47a)  (S47b) |
| N immobilization by microbes | ${FN}_{im, NH4\to BA}=\left[ (VN_{im,NH4}\cdot YN_{g})\cdot BA\cdot NH4 \right]/(K{SN}_{BA1}\cdot\eta)$  ${FN}_{im, NO3\to BA}=\left[ \left( VN_{im,NO3}\cdot YN_{g} \right)\cdot BA\cdot NO3 \right]/(K{SN}_{BA2}\cdot\eta)$  $\eta=1+\frac{BA}{K{SN}_{BA1}}+\frac{NH4}{K{SN}_{BA1}}+\frac{NO3}{K{SN}_{BA2}}+\frac{NH4}{K{SN}_{VG1}}+\frac{NO3}{K{SN}_{VG2}}$ | (S48)  (S49)  (S50) |
| N uptake by plants | ${FN}_{im, NH4\to VG}=\left[ \left( VN_{VG,NH4}\cdot rGPP \right)\cdot NH4 \right]/(K{SN}_{VG1}\cdot\eta)$  ${FN}_{im, NO3\to VG}=\left[ \left( VN_{VG,NO3}\cdot rGPP \right)\cdot NO3 \right]/(K{SN}_{VG2}\cdot\eta)$  Coefficient *rGPP* =*f*(GPP), e.g., $rGPP=exp[\omega_{VG}\cdot\left( GPP/GPP_{ref}-1 \right)]$ | (S51)  (S52) |
| NH_4_^+^ sorption | $NH4=\left[ A+\sqrt{A^{2}+4K_{ba,NH4}\cdot NH4tot} \right]/{(2{\cdot K}_{ba,NH4})}$  $NH4ads=NH4tot-NH4$  where $A=K_{ba,NH4}\cdot\left( NH4tot-{NH4}_{max} \right)-1$  derived from: $NH4ads+NH4=NH4tot$  $NH4ads={NH4}_{max}\cdot\left( K_{ba,NH4}\cdot NH4 \right)/(1+K_{ba,NH4}\cdot NH4)$ | (S53a)  (S53b)  (S53c)  (S53d)  (S53e) |
| NO_3_^–^ and NO_2_^–^ leaching | $FN_{leach,NO3}=NO3\cdot{fN}_{leach}$  $FN_{leach,NO2}=NO2\cdot{fN}_{leach}$  where: $fN_{leach}=r_{leach}\cdot fN_{dissolved}\cdot\left( {\theta_{perc}}/\theta\right)$  ${fN}_{dissolved}=\left( \theta/{\theta_{sat}} \right)^{3}$  $\theta_{perc}=\theta_{excess}\cdot\left[ 1-\exp\left( -{\Delta t}/{TT_{perc}} \right) \right]$  $\theta_{excess}=max(0,\theta-\theta_{FC})$  $TT_{perc}={\left( \theta_{sat}-\theta_{FC} \right)\cdot Depth}/{K_{sat}}$  $r_{leach}\in(0,1)$: scaling factor;  $\theta$: volumetric soil water content (SWC); $\theta_{sat}$ and $\theta_{FC}$: SWC at saturation and field capacity; *Depth*: soil depth (cm); $\theta_{excess}$: excess SWC available for percolation; $\theta_{perc}$: SWC percolation; $K_{sat}$: saturated hydraulic conductivity (cm h^–1^); $\Delta t$: given time-period (= 1h); $TT_{perc}$: travel time for percolation (h) | (S54a)  (S54b)  (S54c)  (S54d)  (S54e)  (S54f)  (S54g) |
| NO, N_2_O, N_2_ gas emission: *j* = 4,5,6 | $FN_{emit_{j}}=\left[ Ds_{j}\cdot\left( N_{j}-Nair_{j} \right)/\left( 0.5\cdot Depth \right) \right]/{Depth}$  where: $Ds_{j}$: gas diffusivity in soil (cm^2^ h^–1^);  $N_{j}$ and $Nair_{j}$ gas concentration in soil and air (mg N cm^–3^) | (S55) |

## Table S6. MEND model parameters

| **ID** | **Parameter** | **Description** | **Range** | **Value** | **Units** |
| --- | --- | --- | --- | --- | --- |
| 1 | *LF_0_* | Initial fraction of *PO*, *LF_0_* = *PO*/(*PO*+*PH*) | (0.1, 1.0) | 0.1 | — |
| 2 | *r_0_* | Initial active fraction of microbes | (0.01, 1) | 0.01 | — |
| 3 | *fR_a_* | Scaling factor for autotrophic respiration (*R_a_*) | (0.1, 0.4) | 0.2 | — |
| 4 | *fINP* | Scaling factor for litter input rate | (0.1, 0.9) | 0.3 | — |
| 5 | *VP* | Maximum specific decomposition rate | (0.1, 100) | 50 | mg C mg^−1^ C h^−1^ |
| 6 | *K_P_*_O_ | Half-saturation constant (HSC) for *PO* decomposition | (40,100) | 60 | mg C cm^−3^ soil |
| 7 | *fK_M_* | *K_M_* = *K_P_*_O_×*fK_M_*, *K_P_*_H_ = *K_P_*_O_/*fK_M_*  *K_P_*_H_ and *K_M_* are HSC for *PH* and *M*, respectively | (2, 20) | 10 | — |
| 8 | *Q*_max_ | Maximum sorption capacity | (0.5, 5.0) | 1.5 | mg C cm^−3^ soil |
| 9 | *K_ba_* | Binding affinity | (1, 16) | 6 | (mg C cm^−3^ soil) ^−1^ |
| 10 | *k_des_* | Desorption rate for DOM | (1e-4, 0.01) | 0.006 | mg C cm^−3^ soil h^−1^ |
| 11 | *r_E_* | Enzyme turnover rate | (1e-4, 0.01) | 1.23e-4 | mg C mg^−1^ C h^−1^ |
| 12 | *p_EP_* | [*V_m_*×*p_EP_*] is the production rate of *EP* (*EPO* + *EPH*), *V_m_* is the specific maintenance rate for *BA* | (1e-3, 0.1) | 1.48e-3 | — |
| 13 | *fp_EM_* | *fp_EM_* = *p_EM_*/*p_EP_*, [*V_mt_*×*p_EM_*] is the production rate of *EM* | (0.1, 5.0) | 4.5 | — |
| 14 | *f_D_* | Fraction of decomposed *PO* and *PH* allocated to *D* | (0.05, 1) | 0.75 | — |
| 15 | $g_{D}$ | Fraction of dead *BA* allocated to *D* | (0.01, 1) | 0.5 | — |
| 16 | $g_{PO}$ | $\left( 1-g_{D} \right)\cdot g_{PO}$ is the fraction of dead *BA* entering *PO* | (0.05, 0.2) | 0.1 | — |
| 17 | *V_g_* | Maximum specific uptake rate of *D* for growth | (1e-3, 0.1) | 0.004 | mg C mg^−1^ C h^−1^ |
| 18 | *α* | *= V_m_* /( *V_g_* + *V_m_*), *V_m_* is max specific maintenance rate | (0.01, 0.5) | 0.05 | — |
| 19 | *K_D_* | HSC for microbial uptake of *D* | (1e-4, 0.5) | 1e-4 | mg C cm^−3^ soil |
| 20 | *Y_g_*(*T*_ref_) | Intrinsic C use efficiency at reference temperature (*T*_ref_) | (0.2, 0.4) | 0.2 | — |
| 21 | *kY_g_* | Slope for *Y_g_* dependence of temperature | (1e-3, 0.016) | 0.005 | 1/°C |
| 22 | *Q*_10_ | Q_10_ for temperature response function | (1.2, 2.5) | 1.8 | — |
| 23 | *γ* | Max microbial mortality rate = *V_m_*× *γ* | (0.01, 20) | 0.01 | — |
| 24 | *β* | Ratio of dormant maintenance rate to *V_m_* | (5e-4, 0.05) | 0.001 | — |
| 25 | *ψ_A2D_* | Soil water potential (SWP) threshold for microbial dormancy; both *ψ_A2D_* & *ψ_D2A_* < 0 | (–0.6, –0.2) | 0.46 | MPa |
| 26 | *τ* | *ψ_D2A_* = *ψ_A2D_* × *τ*, *ψ_D2A_* is the SWP threshold for microbial resuscitation | (0.1, 0.9) | 0.39 | — |
| 27 | *ω* | Exponential in SWP function for microbial dormancy or resuscitation | (1, 6) |  | — |
| *Inorganic nitrogen parameters* | | | | | |
| 28 | $\boldsymbol{V}\boldsymbol{N}_{\boldsymbol{im,BA}}$ | Max specific microbial N immobilization rate | (1e-4, 0.1) | **Calibrated** | mg N mg^−1^ C h^−1^ |
| 29 | $K{SN}_{BA1}$ | HSC for microbial immobilization of NH_4_^+^ | (1e-4, 0.01) | 1.8e-4 | mg N cm^−3^ soil |
| 30 | $K{SN}_{BA2}$ | HSC for microbial immobilization of NO_3_^–^ | (1e-4, 0.01) | 4.1e-4 | mg N cm^−3^ soil |
| 31 | $\boldsymbol{VNit}$ | Max specific nitrification rate (*VN*_1_) | (0.1, 1000) | **Calibrated** | mg N mg^−1^ C h^−1^ |
| 32 | $\boldsymbol{V}\boldsymbol{N}_{\boldsymbol{denit}}$ | Max specific denitrification rate | (1e-4, 1.0) | **Calibrated** | mg N mg^−1^ C h^−1^ |
| 33 | $\boldsymbol{VNif}$ | Max specific N fixation rate (*VN*_6_) | (1e-4, 0.1) | **Calibrated** | mg N mg^−1^ C h^−1^ |
| 34 | *KSN*_1_ | HSC for nitrification | (1e-3, 1.0) | 0.0012 | mg N cm^−3^ soil |
| 35 | *KSN*_2_ | HSC for denitrification of NO_3_^–^ and NO_2_^–^ | (1e-4, 0.1) | 0.0018 | mg N cm^−3^ soil |
| 36 | *KSN*_4_ | HSC for denitrification of NO and N_2_O | (1e-4, 0.1) | 0.0018 | mg N cm^−3^ soil |
| 37 | *KSN*_6_ | HSC for N fixation | (1e-4, 0.1) | 0.1 | mg N cm^−3^ soil |
| 38 | $\boldsymbol{V}\boldsymbol{N}_{\boldsymbol{VG}}$ | Max plant N uptake rate | (1e-6, 1e-3) | **Calibrated** | mg N cm^−3^ h^−1^ |
| 39 | *KSN*_VG1_ | HSC for plant uptake of NH_4_^+^ | (1e-4, 0.01) | 0.0012 | mg N cm^−3^ soil |
| 40 | *KSN*_VG2_ | HSC for plant uptake of NO_3_^–^ | (1e-4, 0.01) | 0.0018 | mg N cm^−3^ soil |
| 41 | *ω_VG_* | Exponential for calculating *rGPP* as a function of GPP | (0.01, 1) | 0.5 | — |
| 42 | *NH4*_max_ | Maximum sorption capacity for NH_4_^+^ | (1e-5, 0.01) | 0.0057 | mg N cm^−3^ soil |
| 43 | *K_ba,NH4_* | Binding affinity for NH_4_^+^ | (1, 1e4) | 100 | (mg N cm^−3^ soil) ^−1^ |
| 44 | *r_leach_* | Scaling factor for NO_3_^–^ and NO_2_^–^ leaching | (0.01, 1) | 0.02 | — |

## Table S7. Response variables for model calibration and performance evaluation. The three allocation scenarios (A0, A1, and A2) are described in Supplementary Table S1. The objective functions are presented in Supplementary Section 1.4.

| **Response variable** | **Description** | **Objective function** | **Data points** | **fOBJ_i_** | | |
| --- | --- | --- | --- | --- | --- | --- |
|  |  |  |  | A0 | A1 | A2 |
| NH_4_^+^ | Ammonium concentration | *J_1_* = 0.8\|*PBIAS*\| + 0.2*MARE* | 8 | 0.13 | 0.10 | 0.13 |
| NO_3_^–^+NO_2_^–^ | Nitrate + Nitrite concentration | *J_2_* = 0.8\|*PBIAS*\| + 0.2*MARE* | 8 | 0.17 | 0.17 | 0.17 |
| N_mn-net_ | Net N mineralization flux | *J_3_* = *MAREt*, tolerance = 0.5 | 10 | 0 | 0 | 0 |
| N_nit_ | Nitrification flux | *J_4_* = *MAREt*, tolerance = 0.9 | 10 | 0 | 0 | 0 |
| N_im,VG_ | Plant N uptake flux | *J_5_* = *MAREt*, tolerance = 0.5 | 1 | 0 | 0 | 0 |
| N_fix_ | N fixation flux | *J_6_* = *MAREt*, tolerance = 0.2 | 1 | 0 | 0 | 1 |
| fOBJ (best=0) | — | $J=\sum w_{i}\times J_{i}$ | — | 0.10 | 0.09 | 0.26 |

**Notes:** *PBIAS* is percent bias; *MARE* is the mean absolute relative error; *MAREt* is the *MARE* with a tolerance; *OBJ* is the objective function value, which the best value is 0; $w_{i}$ is the weighting factor; A0, A1, and A2 are three allocation scenarios.

# 3. Supplementary results

## 3.1 Time series of the relative saturation level of a substrate


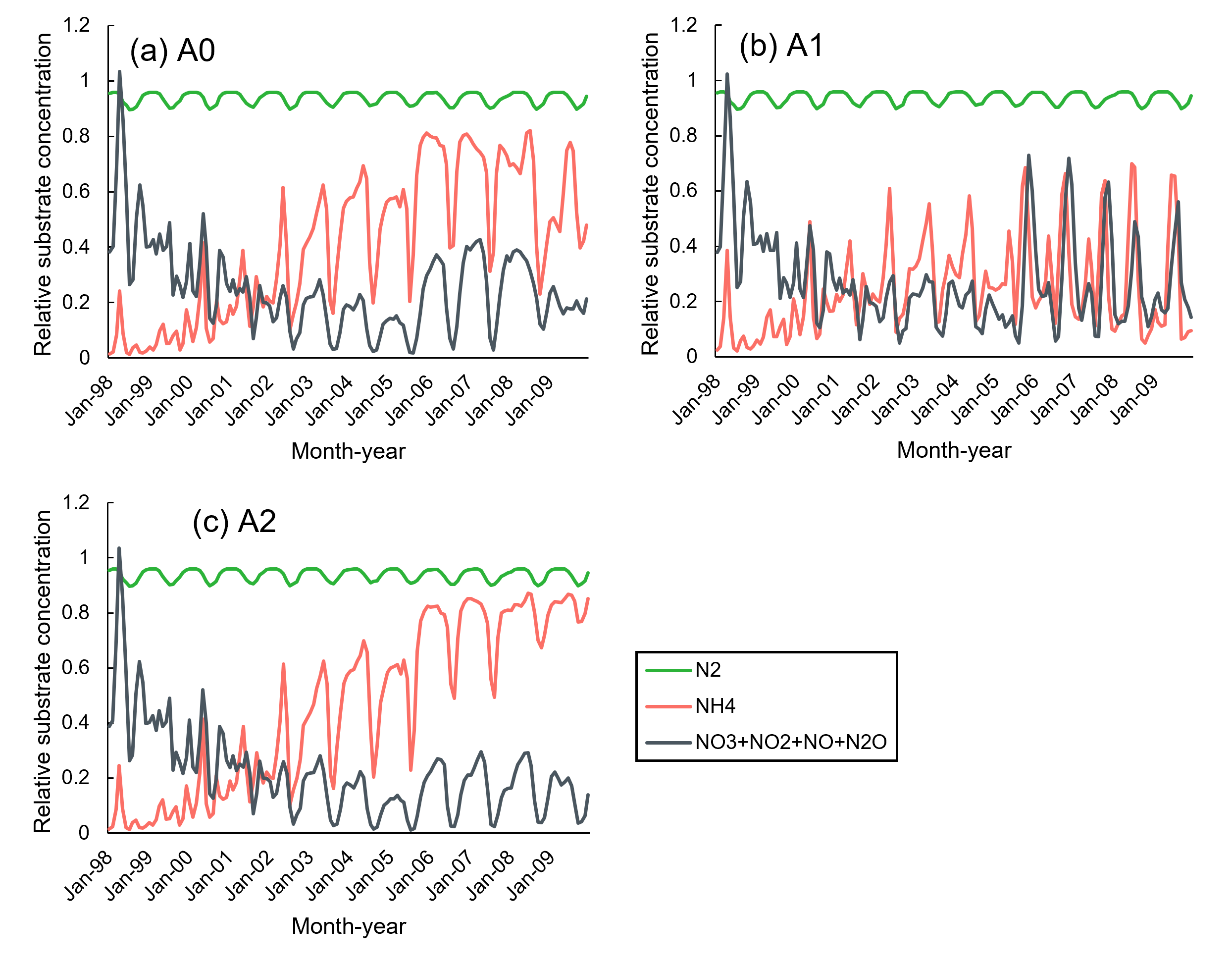


**Figure S3.** Time series of the relative substrate concentration for the three enzyme allocation scenarios: A0, A1, and A2 (defined as in Table S1). The relative substrate concentration is defined as $N_{i}/({KsN}_{i}+N_{i})$, where $i=1-6$ indicated the substrate of N_2_, NH_4_^+^, NO_3_^–^, NO_2_^–^, NO, and N_2_O, respectively.

## 3.2 Uncertainty quantification

The uncertainties in model parameters and output variables are evaluated by the Uncertainty Quantification by Critical Objective Function Index (UQ-COFI) method [12], which is based on a global stochastic optimization technique (e.g., SCE in this study). The confidence region of parametric space were determined by selecting those parameter sets resulting in objective function values ($J$) less than the COFI value ($J_{cr}$) from the feasible parameter space. The COFI ($J_{cr}$) is defined as:

| $J_{cr}=J_{opt}\cdot\left( 1+\frac{p}{n-p}\cdot F_{\alpha, p,n-p} \right)$ | (S61) |
| --- | --- |

where$J_{cr}$ is the COFI that defines the parameter uncertainty region, $J_{opt}$ is the optimum (minimum) objective function value that is calculated by Eq. S56, *n* is the number of measured data points, *p* is the number of parameters, and *F_α,p,n−p_* is the value of the F-distribution for *α*, *p*, and *n−p*. It is evident that more observed data points (i.e., larger *n*) and less undetermined parameters (i.e., smaller *p*) would reduce parametric uncertainty (i.e., lower $J_{cr}$).

The procedure of UQ-COFI includes: (i) implementing the SCE algorithm with multiple different random seeds to search ‘relatively optimal’ parameter sets that minimizing the objective function ($J$ in Eq. S56); (ii) collecting the optimal parameter set generated in each loop of the SCE searching process to form a feasible parameter space; (iii) determining the critical objective function index (COFI) ($J_{cr}$) based on $J_{opt}$ (minimum $J$ value), *n* (number of measurements) and *p* (number of model parameters); (iv) constructing the parametric surface of the confidence space by selecting those parameter sets resulting in $J$ ≤ $J_{cr}$ from the feasible parameter space; (v) conducting model simulations using these selected parameter sets; and (vi) quantifying the uncertainty in model output variables.

To make the uncertainty results comparable among different scenarios, we compute the relative uncertainty (ReUn) [27] of an output variable in response to variations in model parameters:

| $ReUn=\frac{{Width}_{90\%CI}}{Mean}$ | (S62) |
| --- | --- |

where ${Width}_{90\%CI}$ denotes the width of the 90% confidence interval, and $Mean$ is the mean value.

In this study, we focus on the ReUn of two output variables: total inorganic N flux (Flux-Ninorg) and total production of N-related enzymes (Enz-Ninorg).


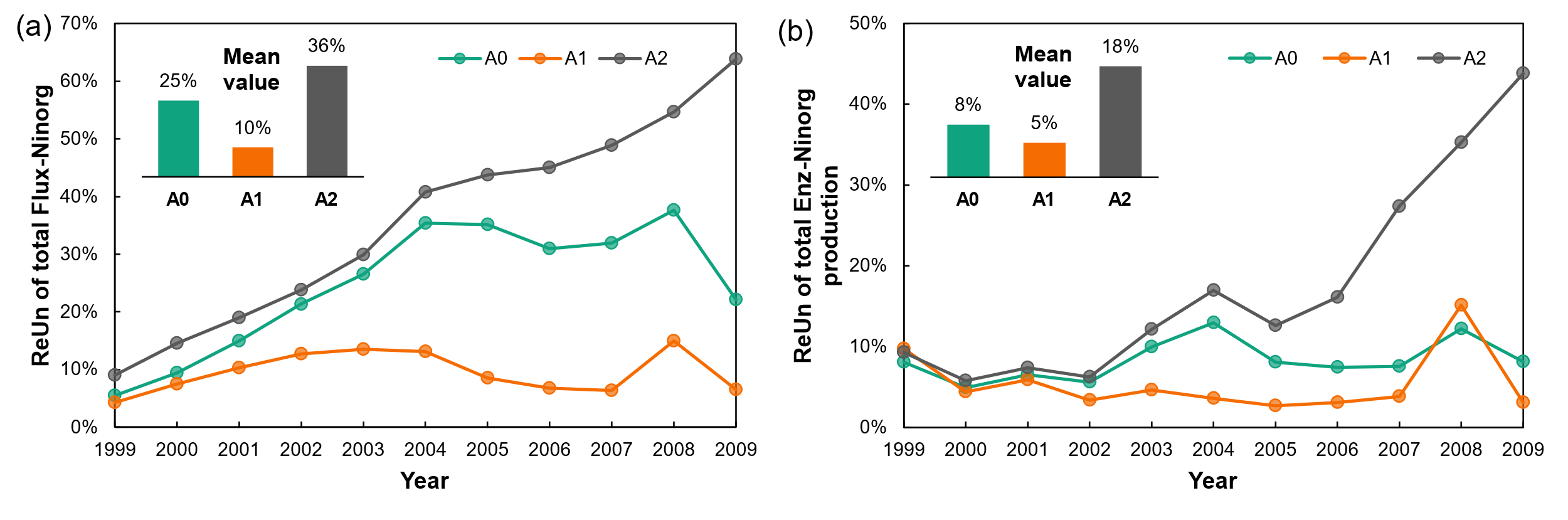


**Figure S4. Relative uncertainty (ReUn) of the N-dynamics component.** (a) Total inorganic N flux. (b) Total production of enzymes catalyzing inorganic N reactions. ReUn is defined as the ratio of 90% confidence intervals to the mean value.

# References

1. Wang G, Gao Q, Yang Y, Hobbie SE, Reich PB, Zhou J. Soil enzymes as indicators of soil function: A step toward greater realism in microbial ecological modeling. Glob Chang Biol. 2022;28(5):1935-50.

2. Fanin N, Fromin N, Barantal S, Hättenschwiler S. Stoichiometric plasticity of microbial communities is similar between litter and soil in a tropical rainforest. Scientific Reports. 2017;7(1):12498.

3. Mooshammer M, Wanek W, Zechmeister-Boltenstern S, Richter A. Stoichiometric imbalances between terrestrial decomposer communities and their resources: mechanisms and implications of microbial adaptations to their resources. Front Microbiol. 2014;5:22.

4. Mooshammer M, Wanek W, Hämmerle I, Fuchslueger L, Hofhansl F, Knoltsch A, et al. Adjustment of microbial nitrogen use efficiency to carbon:nitrogen imbalances regulates soil nitrogen cycling. Nature Communications. 2014;5(1).

5. Schimel JP, Weintraub MN. The implications of exoenzyme activity on microbial carbon and nitrogen limitation in soil: a theoretical model. Soil Biology and Biochemistry. 2003;35(4):549-63.

6. Averill C. Divergence in plant and microbial allocation strategies explains continental patterns in microbial allocation and biogeochemical fluxes. Ecol Lett. 2014;17(10):1202-10.

7. Wang G, Huang W, Mayes MA, Liu X, Zhang D, Zhang Q, et al. Soil moisture drives microbial controls on carbon decomposition in two subtropical forests. Soil Biology and Biochemistry. 2019;130:185-94.

8. Wang G, Post WM, Mayes MA, Frerichs JT, Sindhu J. Parameter estimation for models of ligninolytic and cellulolytic enzyme kinetics. Soil Biology and Biochemistry. 2012;48:28-38.

9. Frey SD, Lee J, Melillo JM, Six J. The temperature response of soil microbial efficiency and its feedback to climate. Nature Climate Change. 2013;3(4):395-8.

10. Jian S, Li J, Chen J, Wang G, Mayes MA, Dzantor KE, et al. Soil extracellular enzyme activities, soil carbon and nitrogen storage under nitrogen fertilization: A meta-analysis. Soil Biology and Biochemistry. 2016;101:32-43.

11. Sinsabaugh RL, Belnap J, Findlay SG, Shah JJF, Hill BH, Kuehn KA, et al. Extracellular enzyme kinetics scale with resource availability. Biogeochemistry. 2014;121(2):287-304.

12. Wang G, Jagadamma S, Mayes MA, Schadt CW, Megan Steinweg J, Gu L, et al. Microbial dormancy improves development and experimental validation of ecosystem model. The ISME Journal. 2014;9(1):226-37.

13. Fiencke C, Bock E. Immunocytochemical localization of membrane-bound ammonia monooxygenase in cells of ammonia oxidizing bacteria. Arch Microbiol. 2006;185(2):99-106.

14. Song HS, Thomas DG, Stegen JC, Li M, Liu C, Song X, et al. Regulation-Structured Dynamic Metabolic Model Provides a Potential Mechanism for Delayed Enzyme Response in Denitrification Process. Front Microbiol. 2017;8:1866.

15. Schlesier J, Rohde M, Gerhardt S, Einsle O. A Conformational Switch Triggers Nitrogenase Protection from Oxygen Damage by Shethna Protein II (FeSII). J Am Chem Soc. 2016;138(1):239-47.

16. Coggins SA, Mahboubi B, Schinazi RF, Kim B. Mechanistic cross-talk between DNA/RNA polymerase enzyme kinetics and nucleotide substrate availability in cells: Implications for polymerase inhibitor discovery. J Biol Chem. 2020;295(39):13432-43.

17. Reich PB, Hobbie SE. Decade-long soil nitrogen constraint on the CO2 fertilization of plant biomass. Nature Climate Change. 2013;3(3):278-82.

18. Duan Q, Sorooshian S, Gupta V. Effective and efficient global optimization for conceptual rainfall-runoff models. Water Resources Research. 1992;28(4):1015-31.

19. Wang G, Li W, Wang K, Huang W. Uncertainty quantification of the soil moisture response functions for microbial dormancy and resuscitation. Soil Biology and Biochemistry. 2021;160.

20. Lv A, Qi S, Wang G. Multi-model driven by diverse precipitation datasets increases confidence in identifying dominant factors for runoff change in a subbasin of the Qaidam Basin of China. Science of The Total Environment. 2022;802.

21. Wang G, Jager HI, Baskaran LM, Brandt CC. Hydrologic and water quality responses to biomass production in the Tennessee river basin. GCB Bioenergy. 2018;10(11):877-93.

22. Gao Q, Wang G, Xue K, Yang Y, Xie J, Yu H, et al. Stimulation of soil respiration by elevated CO(2) is enhanced under nitrogen limitation in a decade-long grassland study. Proc Natl Acad Sci U S A. 2020;117(52):33317-24.

23. Mueller KE, Hobbie SE, Tilman D, Reich PB. Effects of plant diversity, N fertilization, and elevated carbon dioxide on grassland soil N cycling in a long-term experiment. Global Change Biology. 2013;19(4):1249-61.

24. Wang G, Chen S. A review on parameterization and uncertainty in modeling greenhouse gas emissions from soil. Geoderma. 2012;170:206-16.

25. Cleveland CC, Townsend AR, Schimel DS, Fisher H, Howarth RW, Hedin LO, et al. Global patterns of terrestrial biological nitrogen (N2) fixation in natural ecosystems. Global Biogeochemical Cycles. 1999;13(2):623-45.

26. Reyes J, Schellberg J, Siebert S, Elsaesser M, Adam J, Ewert F. Improved estimation of nitrogen uptake in grasslands using the nitrogen dilution curve. Agronomy for Sustainable Development. 2015;35(4):1561-70.

27. Xiang D, Wang G, Tian J, Li W. Global patterns and edaphic-climatic controls of soil carbon decomposition kinetics predicted from incubation experiments. Nature Communications. 2023;14(1).
